# All Nations Must Prioritize the COVID-19 Vaccination Program for Elderly Adults Urgently

**DOI:** 10.14336/AD.2021.0426

**Published:** 2021-06-01

**Authors:** Chiranjib Chakraborty, Ashish Ranjan Sharma, Manojit Bhattacharya, Govindasamy Agoramoorthy, Sang-Soo Lee

**Affiliations:** ^1^Department of Biotechnology, School of Life Science and Biotechnology, Adamas University, Barasat-Barrackpore Rd, Kolkata, West Bengal 700126, India.; ^2^Department of Zoology, Fakir Mohan University, VyasaVihar, Balasore 756020, Odisha, India.; ^3^Institute for Skeletal Aging & Orthopedic Surgery, Hallym University-Chuncheon Sacred Heart Hospital, Chuncheon-si, 24252, Gangwon-do, Korea.; ^4^College of Pharmacy and Health Care, Tajen University, Yanpu, Pingtung 907, Taiwan.

**Dear Editor**

A recent editorial by Dr. Nir Barzilai and his colleagues about the geroscience during the period of COVID-19 is highly significant and timely. This article has recommended for clinical trials of therapies likely to geroprotective and implement policies for elderly adults [1].

Aging increases the risk of infection. The elderly population is susceptible to several infectious diseases [2]. In the case of COVID-19, more than millions 139 million of infection and 3.9 million death has been so far recorded. A significant number of deaths occur from the elderly category [3]. Also, age related deterioration of the immune system or immunosenescence is known to be associated with both non-communicable and infectious diseases. This might be the leading cause-related to COVID-19 severity and mortality. In a chronological aging population, biology aging and immunological aging are also significant factors that may affect how they respond to diseases. Hence, along with the chronological aging in the elderly population, there should be a pattern of reaction towards the SARS-CoV-2 infection in terms of biological aging and immunological aging. Therefore, it is essential to decipher the biological aging and immunological aging related mechanism for SARS-CoV-2 infection to minimize the disease in vulnerable senior citizens.

Simultaneously, biological aging and immunological aging may play a significant role in contributing to the severity of COVID-19 outcome and mortality towards the elderly population [4, 5]. But it is yet to be investigated thoroughly. Therefore, research is urgently needed to understand the three trajectories and their relations with clinical manifestation of the height severity COVID-19 outcome in the elderly population. Simultaneously, the changes in adaptive and innate immunity against the viral infection in older adults need to unfold with more details. In a recent article, Koff and Williams have suggested to explore the immune system pattern among the world’s most vulnerable population against the pandemic [6].

Currently, the COVID-19 vaccination has been rolled out in most of the countries worldwide. Along with the general population, vaccination is also started for the older population in each country. The United Nations report has estimated that the global elderly population (>60 years of age) to be 962 million in 2017. In Africa, the elderly population's growth was fastest with 69 million in 2017. The second growing region for the elderly population was Latin America and the Caribbean. The elderly population in Asia, Europe, and Northern America were estimated at 549 million, 183 million, and 78 million respectively in 2017 (www.un.org/en/development/desa/population/publications/pdf/ageing/WPA2017_Highlights.pdf). This population is growing rapidly in all countries, and therefore they need to be vaccinated against the COVID-19 as a priority basis.

Different vaccination programs were recommended for the elderly for several diseases, were launched from time to time in Europe and the USA. These two regions are actively vaccinating against several target diseases for the aging adults such as pneumococcal infection, seasonal influenza, group B streptococcus, varicella zoster virus, etc. Due to immunosenescence, the elderly population is more vulnerable to diseases. At the same time, immunosenescence is one of the major problems of vaccination for elderly. Due to this issue, a decrease in the adaptive immune system's efficiency was noted effectively, such as the production rate of naïve T cells and B cells, quality, and composition of the mature lymphocyte pool, which can restrict the efficacy of vaccines in elderly individuals [7].

**Figure 1.**
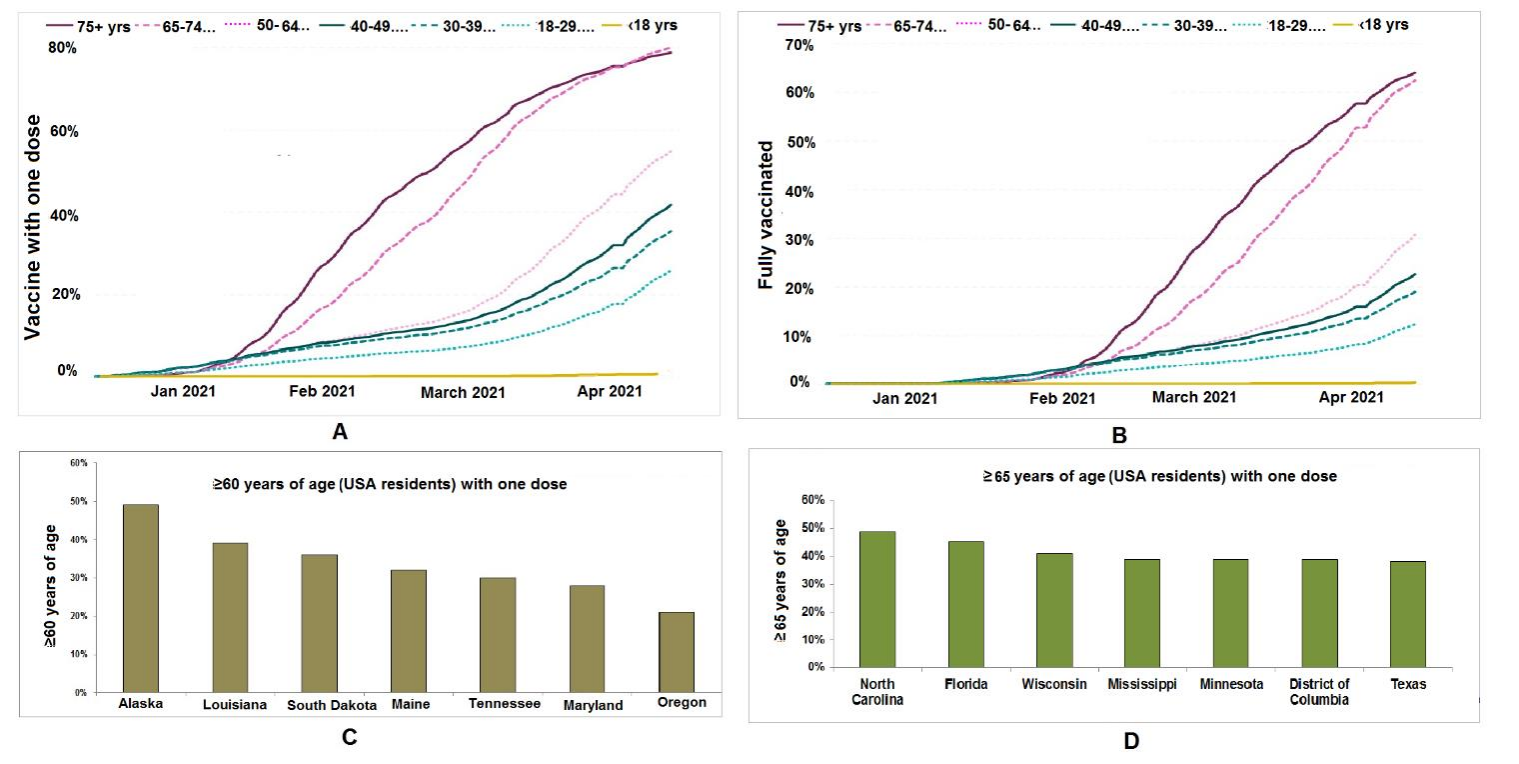
Mapping of the COVID-19 vaccination from for elderly individual in the USA. (A) Age-wise COVID-19 vaccination up to 1^st^ week of April 2021 in the USA who received at least one dose (B) Age-wise COVID-19 vaccination up to 1^st^ week of April 2021 in the USA who are fully vaccinated (C) State-wise COVID-19 vaccination to the elderly individuals (>60 years of age) in the USA who received at least one dose (D) State-wise COVID-19 vaccination to the elderly individuals (>65 years of age) in USA who received at least one dose (Data source: CDC, USA source (Fig. 1A and B) and https://www.kff.org (Fig. 1C and D).

Approximately, 13 COVID-19 vaccines have received approval at least in one country as of late March 2021 that include Pfizer-BioNTech (BNT162b), Moderna (mRNA-1273), BBIBP-CorV, Oxford-AstraZeneca (ChAdOx1 nCoV-19), CoronaVac, CoviVac, Covaxin, Sputnik V, Johnson & Johnson, Convidicea, RBD-Dimer, and EpiVacCorona. Different countries are using these vaccines, and most of them are informed about their efficacy. However, we need to understand the efficacy of these vaccines and the antibody responses in the elderly populations particularly. A study in two hospitals in the UK found that two vaccines (Oxford AstraZeneca and Pfizer BioNTech) can prevent 80% of the hospitalizations of senior citizens above the age of 80 [8]. However, most developed countries have started to vaccinate the elderly population with the general population. Till early April 2021, more than 60% of elderly population (>65 years of age) were fully vaccinated in the USA. About 80% of the elderly (>65 years of age) were fully vaccinated (Fig. 1). The UK has started a nationwide vaccination program giving the first shot to the super-seniors above the age of 80 (www.npr.org/sections/coronavirus-live-updates/2020/12/08/944125280/u-k-begins-nationwide-coronavirus-immunization-largest-in-nations-history).

It has been observed that there is a limited supply of COVID-19 vaccines in every country. Every country makes a vaccine distribution policy immediately, describing how the government will deal with the limited supply of COVID-19 vaccine among the population and how they will distribute it among the people. In this case, target population selection for the COVID-19 vaccination program is a significant factor. Therefore, it is necessary to understand the high-risk populations that should be vaccinated first [9]. Consequently, the target population for the first immediate vaccination group should include older people with comorbidity, other older adults, clinicians, nurses, comorbid people, front line workers, emergency and essential service staff, etc. However, older people with comorbidity and other older people are more vulnerable and should be vaccinated at the earliest. At the same time, every country should focus on more vaccine production in a limited time frame. Government should be more dedicated to increasing vaccine production several folds. Any country with limited vaccine production capacity should join hands with the vaccine-producing country and invest in COVID-19 vaccine production. Government efforts for revamping the production and distribution system could help vaccinate the whole country's population as early as possible.

At the same time, all nations should frame a policy independently and immediately to vaccinate their elderly population. The low and middle-income countries should vaccinate all the elderly because this population may be affected by co-morbid conditions. Simultaneously, high-income nations should help the low-income nations to enforce a successful COVID-19 vaccination program for the elderly. All countries should take rapid action to support the elderly to enhance their longevity.
